# The promise of computer-assisted auscultation in screening for structural heart disease and clinical teaching

**DOI:** 10.5830/CVJA-2012-007

**Published:** 2012-08

**Authors:** L Zühlke, L Myer, BM Mayosi

**Affiliations:** School of Adolescent and Child Health, Red Cross War Memorial Children’s Hospital, and Department of Medicine, University of Cape Town, Cape Town, South Africa; School of Public Health and Family Medicine, University of Cape Town, Cape Town, South Africa; Department of Medicine, Groote Schuur Hospital and University of Cape Town, Cape Town, South Africa

**Keywords:** auscultation, screening for cardiac disease, clinical teaching, primary healthcare

## Abstract

**Abstract:**

Cardiac auscultation has been the central clinical tool for the diagnosis of valvular and other structural heart diseases for over a century. Physicians acquire competence in this technique through considerable training and experience. In Africa, however, we face a shortage of physicians and have the lowest health personnel-to-population ratio in the world. One of the proposed solutions for tackling this crisis is the adoption of health technologies and product innovations to support different cadres of health workers as part of task shifting.

Computer-assisted auscultation (CAA) uses a digital stethoscope combined with acoustic neural networking to provide a visual display of heart sounds and murmurs, and analyses the recordings to distinguish between innocent and pathological murmurs. In so doing, CAA may serve as an objective tool for the screening of structural heart disease and facilitate the teaching of cardiac auscultation. This article reviews potential clinical applications of CAA.

## Abstract

Prior to the development of echocardiography and other imaging modalities, the stethoscope was the central investigative tool for the diagnosis of structural heart disease. While the stethoscope is relatively inexpensive and widely available, it remains a qualitative and subjective method of evaluating heart sounds, murmurs and other cardiac noises. The development of the digital stethoscope with additional analysis software promises to transform the stethoscope into a tool for quantitative and objective clinical evaluation of the heart. Such a tool may improve the assessment of innocent murmurs, reduce observer variation due to human acoustic abilities, and facilitate the teaching of cardiac auscultation.

## The assessment of innocent murmurs

Up to 80% of paediatric patients have a cardiac murmur, although less than 1% will eventually have a pathological condition underlying the murmur.^1^ Praecordial murmurs are also common among young adults, occurring in between 29 and 52% of the general population.^2^ The ability to distinguish between an innocent and a pathological murmur is therefore a fundamental clinical skill that should be imparted to doctors and other healthcare professionals involved in the screening and diagnosis of heart disease.

The innocent murmur is, however, the most frequently misdiagnosed condition when testing auscultation skills of medical practitioners.^3^ As a consequence, large numbers of patients with an innocent murmur are inappropriately referred for echocardiography, which has serious economic implications, in addition to causing undue concern among healthy individuals and their families.^4^

Echocardiography is the first-line imaging modality for the confirmation of a diagnosis of structural heart disease.^5^ The American Heart Association and American College of Cardiology define a class 1 recommendation for echocardiography as: ‘where clinical features indicate at least a moderate probability that a murmur reflects structural heart disease’.^6^

These guidelines discourage the indiscriminate use of echocardiography as a screening tool due to the cost and the potential for overdiagnosis of disease. Despite this, patients are still referred inappropriately for echocardiography for the evaluation of innocent murmurs. In a retrospective review of 3 460 adult referrals for echocardiogram with the coding ‘murmur’ as the primary reason for referral, less than 50% had significant valvular disease.^7^ A study in Norway showed that only 10% of children referred to a cardiac centre for investigation of a cardiac murmur were subsequently found to have a congenital cardiac lesion.^8^ The majority of these children (71%) were referred by general practitioners, though in only 17% was a diagnosis made by the referring physician.

There is therefore a major need to improve the ability of medical practitioners to identify murmurs that have a high probability of structural heart disease. CAA promises to address this need by providing a decision support tool on the likelihood of the presence of pathological murmur.

## CAA as a teaching aid for cardiac auscultation

In the past decade, numerous reports have expressed concern over the training of healthcare professionals in cardiac auscultation.^3^,^9^,^10^ Despite the fact that directors of USA-based medical school programmes interviewed considered auscultation an important clinical skill, only 27% of internal medicine and 37% of cardiology programmes offered any structured teaching of auscultation.^10^ A multi-centre study testing medical students, trainees, physicians and teaching faculty more comprehensively demonstrated low ability to recognise systolic and diastolic murmurs.^11^ These studies indicate the need for more directed teaching of cardiac auscultation.

The ability of a digital stethoscope to record sounds, replay them at different speeds, provide a visual display and develop a database of heart sounds for on-going review creates a unique vehicle for teaching auscultation and is a major incentive for the addition of CAA into current teaching programmes.

## The development of computer-assisted auscultation

The pursuit of a quantitative and objective stethoscope has occupied investigators for decades, and electronic stethoscopes have been available commercially for some time.^12^ Early criticisms related to difficulty of use, distortion of sound and cumbersome designs. However, over the past decade, rapid advances have been made not only in the electronic stethoscopes themselves, but also in the associated computer analysis.^13^

**Fig. 1.**
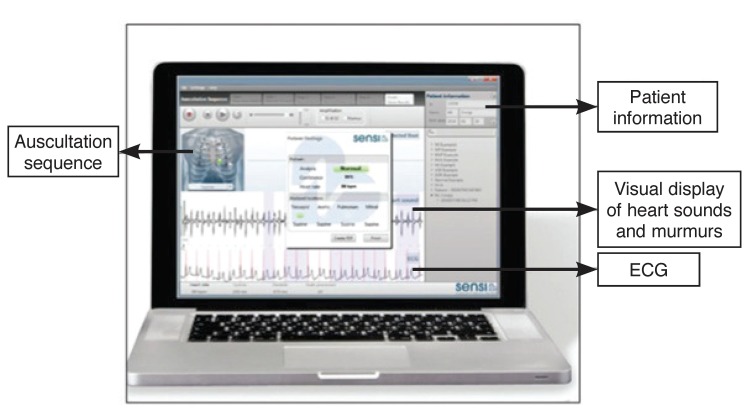
The computer interface, as displayed on a laptop computer, depicts the areas of auscultation, visual display of heart sounds and murmurs, as well as ECG. (Reproduced with the permission of Mr Thys Cronje.)

**Fig. 2.**
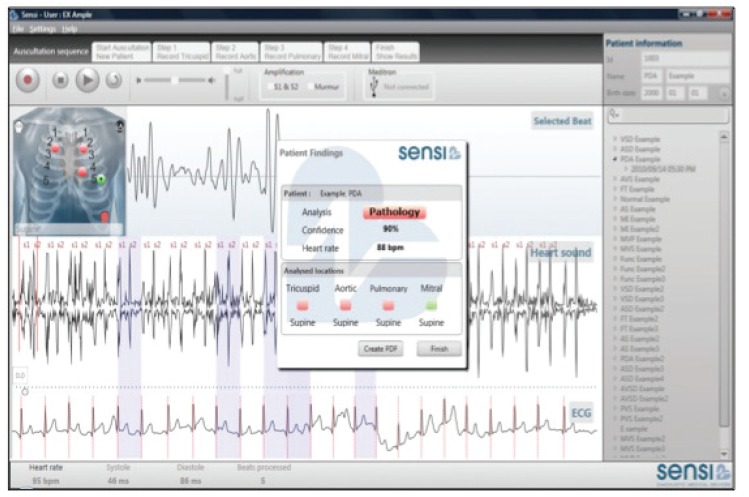
In this display, analysis has determined that pathological murmurs were detected in the tricuspid, aortic and pulmonary areas. The computer interface also displays the level of confidence (90%) and the heart rate. (Reproduced with the permission of Mr Thys Cronje.)

The CAA provides a spectral and temporal analysis of heart sounds and a graphic display of the energy profiles relating to systolic and diastolic murmurs (Fig. 1).^14^ The quantitative measurement of the intensity of the heart sounds and murmurs in the spectral display, which is recorded simultaneously with the waveform of the sounds, allows objective classification into normal and abnormal sounds (Fig. 2).^15^ Signals obtained electronically may be subjected to objective visual and numerical analysis, transmitted to distant sites, and stored for medical and research purposes.

The Food and Drug Administration (FDA) has approved the first CAA system as an aide to the physician for the detection of abnormal heart murmurs. Using this software, the sensitivity for detection of murmurs increased from 77 to 89%, while the referral sensitivity increased from 87 to 93% among primary-care physicians.^16^ Another decision support system, aimed at the paediatric population and utilising novel signal-processing techniques, recently reported a specificity of 94% and a sensitivity of 91% for the detection of murmurs associated with structural heart disease.^17^,^18^

## Opportunities for clinical application

CAA is a promising decision support tool for the identification of pathological murmurs and appropriate referral of cases for further investigation. Primary-care physicians assessing asymptomatic patients with heart murmurs were able to increase the sensitivity of pathological murmurs detected with the use of CAA from 77 to 89%, while referral sensitivity increased from 87 to 93%, and specificity increased from 64 to 79%.^16^

The clinical utility of the digital stethoscope and associated software in detecting cardiac disease has great potential (Table 1). A recent article described an autonomous auscultation system which could be used to screen for cardiovascular disease in the rural areas of Africa.^19^ The performance of the system was impressive, with a sensitivity of 82% and a specificity of 88% for the identification of pathological murmurs, demonstrating its potential benefit as a screening tool in a rural healthcare environment.

**Table 1.** Clinical Uses For CAA

Screening for structural heart disease	Screening for structural heart disease at primary healthcare level can be improved by the additional of an objective tool to aid referral decision making. Areas where this could potentially be of application include pre-athletic screening, antenatal screening of pregnant women, and screening for heart failure and early rheumatic heart disease in asymptomatic people.
Clinical teaching of cardiac auscultation	CAA may aid in more directed teaching in cardiac auscultation. This includes specific training in auscultation sequence, length of auscultation at each site, as well in the creation of data banks of auscultation recordings. Sounds can be relayed to multiple listeners simultaneously, replayed at different speeds and a visual display can improve retention of information. CAA can also be incorporated into distance-learning programmes.
Task shifting and telemedicine	The global shortage of trained medical specialists requires the training of a new cadre of mid-level and community health workers who may benefit from the use of CAA as a decision support tool in screening for structural heart disease. Telemedicine may be used to link these cadres of health workers, who are often in remote areas, to clinicians and secondary and tertiary centres.

The need for a method to detect athletes at risk of sudden cardiac death due to hypertrophic obstructive cardiomyopathy led to a pilot study examining the level of agreement between auscultation by a cardiologist and the results of CAA.^20^ This study was able to identify ejection systolic murmurs that are louder in standing than in reclining positions: a cardinal sign of hypertrophic obstructive cardiomyopathy.

The third heart sound is an early abnormality in adult patients with heart failure. It is frequently missed in a cursory examination of the heart, but is easily recorded by means of CAA.^21^

The most common cause of acquired heart disease in the world, rheumatic heart disease, has long been neglected due to its waning incidence in the developed world.^22^ A landmark study in Mozambique and Cambodia, however, demonstrated an almost 10-fold under-appreciation of affected patients, using conventional auscultation compared to portable echocardiography.^23^ It is currently being investigated whether CAA improves the early identification of pathological heart sounds in patients with rheumatic heart disease.^24^

Co-existing cardiovascular disease during pregnancy is a leading cause of maternal mortality and contributes to significant morbidity.^25^ Early identification of women with cardiac disease is essential to managing the effects of cardiac disease during and after pregnancy. Certain conditions such as rheumatic mitral stenosis are poorly tolerated in pregnancy. It is likely that mitral stenosis and other structural heart diseases would be identified during routine antenatal screening of pregnant women in primary healthcare clinics if CAA was used as a decision support tool for nurses and doctors in the antenatal clinic.

## The use of CAA in task shifting and telemedicine

A recent World Health Organisation report outlined the crisis in human resources in healthcare, and focused on the particular shortage of trained specialists in sub-Saharan Africa.^26^ This shortage has resulted in the shifting of tasks normally performed by specialists, to mid-level and community health workers.^27^ The use of technology to support community health workers has been recommended, as many other areas of medicine have benefitted by incorporating technology to identify disease in remote areas of the world.^28^,^29^

A key issue regarding the health worker shortage in sub-Saharan Africa is insufficient training opportunities. Two-thirds of sub-Saharan African countries have only one medical school, and 11 sub-Saharan African countries have no medical school at all.^30^ One of the solutions is the introduction of distance learning and open-access teaching materials.^31^ The ability of the digital stethoscope to transmit banks of recorded data for remote teaching suggests its utility for such a programme. The computer interface also serves as a reminder to students of the correct auscultation method (in terms of placement and length of auscultation).

Another use of CAA is in the field of telemedicine. Recording heart sounds using a digital stethoscope and transmitting the sound data for remote assessment by a cardiologist was demonstrated to have a sensitivity and specificity of 90 and 98%, respectively, for the detection of pathological murmurs, with low inter-observer and intra-observer variability.^32^

## Challenges and further development

Despite the potential of CAA, it has yet to be adopted into mainstream clinical practice. There are several factors which may improve the chances of adoption of this new technology.^33^ Firstly, successful CAA systems must have a clearly defined indication: screening, diagnosis, and/or teaching. In addition, ease of use by personnel is of particular importance. Data must be analysed in real-time and be stored in a format which can be integrated into clinical records. From a technical standpoint, standardised data sets would be of great benefit and need to be highly sensitive and specific.

Finally, single-praecordial-site CAA does not approximate the clinical routine of auscultation in different sites, use of diaphragm, bell and adjunct manoeuvres to comprehensively examine the heart. A new recording device using a multiple-praecordial-site approach, simultaneously acquiring six auscultation sites, a single-channel ECG and a respiratory recording, is under development.

## Conclusion

Heart disease is a major cause of morbidity and mortality in all age groups worldwide. For decades, conventional auscultation has been the mainstay for screening and diagnosis of structural heart disease. However, the inherent limitations of the standard stethoscope have resulted in declining reliance on auscultation findings and the inappropriate referral for echocardiography of large numbers of patients with innocent murmurs.

CAA provides objectivity to a traditionally subjective clinical skill. As an objective diagnostic support tool, it may improve the number of appropriate cases of murmur that are referred for echocardiography from primary care. Furthermore, CAA can provide a new platform for teaching cardiac auscultation to health science students and physicians. Finally, CAA may provide a decision support tool for mid-level and community health workers, with linkage to central expertise and training through telemedicine.
